# A minor tweak in transplant surgery protocols alters the cellular landscape of the arterial wall during transplant vasculopathy

**DOI:** 10.3389/frtra.2024.1260125

**Published:** 2024-04-29

**Authors:** Laura Mickiewicz, Rana Zahreddine, Kévin Cormier, Sophie Peries, Arnaud Del Bello, Muriel Laffargue, Natalia F. Smirnova

**Affiliations:** ^1^ Institute of Metabolic and Cardiovascular Diseases (I2MC), Institut National de la Santé et de la Recherche Médicale (INSERM) U1297, University of Toulouse 3, Toulouse, France; ^2^ CREFRE-Anexplo, Services Phénotypage et Microchirurgie, UMS006, INSERM, Université de Toulouse, UT3, ENVT, Toulouse, France; ^3^ Center for Biological Ressources (Centres de Ressources Biologiques, CRB), IUCT Oncopole, Toulouse University Hospital (CHU de Toulouse), Toulouse, France; ^4^ Department of Nephrology and Organ Transplantation, Toulouse University Hospital (CHU de Toulouse), Toulouse, France

**Keywords:** transplantation, surgery, anastomosis, vascular wall, transplant vasculopathy

## Abstract

**Introduction:**

Transplant vasculopathy (TV) is a major complication after solid organ transplantation, distinguished by an arterial intimal thickening that obstructs the vascular lumen and leads to organ rejection. To date, TV remains largely untreatable, mainly because the processes involved in its development remain unclear. Aortic transplantation in mice, used to mimic TV, relies on highly variable experimental protocols, particularly regarding the type of anastomosis used to connect the donor aorta to the recipient. While the amount of trauma undergone by a vessel can dramatically affect the resulting pathology, the impact of the type of anastomosis on TV in mice has not been investigated in detail.

**Methods:**

In this study, we compare the cellular composition of aortic grafts from BALB/C donor mice transplanted into C57BL/6J recipient mice using two different anastomosis strategies: sleeve and cuff.

**Results:**

While both models recapitulated some aspects of human TV, there were striking differences in the cellular composition of the grafts. Indeed, aortic grafts from the cuff group displayed a larger coverage of the neointimal area by vascular smooth muscle cells compared to the sleeve group. Aortic grafts from the sleeve group contained higher amounts of T cells, while the cuff group displayed larger B-cell infiltrates.

**Discussion:**

Together, these data indicate that a seemingly minor technical difference in transplant surgery protocols can largely impact the cellular composition of the graft, and thus the mechanisms underlying TV after aortic transplantation in mice.

## 1 Introduction

Solid organ transplantation represents the best, if not the only option available for patients with end-stage organ failure (kidney, heart, lung, etc.). With the aging of the population, the number of transplant procedures and of patients on the waiting list for a transplant is steadily increasing (1). While the continuous improvement of immunosuppressive therapies tremendously reduced the rates of acute rejection, chronic rejection, affecting the grafts months or years post-transplant, remains a leading medical challenge (2). Chronic rejection encompasses a set of phenotypes (3), among which transplant vasculopathy (TV), associated with poor prognosis and largely untreatable (4–6).

TV affects the entire arterial tree of a graft, encompassing big arteries and terminal arterioles. TV histologically resembles restenosis, as it displays an obliteration of the vascular lumen, due to the formation of a neointima (NI) rich in vascular smooth muscle cells (VSMCs) (7). Studies on restenosis and atherosclerosis show that neointimal VSMCs derive from dedifferentiated medial VSMCs, which acquire abnormal capacities of migration and proliferation inside the vascular lumen (8). Fewer reports are available on the cellular composition of arteries affected by TV in humans. The existing data show the presence of VSMCs but also recruited cells inside the neointima in the context of TV (9–11).

In an attempt to understand the processes underlying TV, aortic transplantation in mice is a commonly used strategy. Yet it relies on multiple experimental variations throughout the literature: (1) both thoracic and abdominal aortas are used as donor organs (12); (2) they are connected either to the abdominal aorta or the carotid artery of the recipient (13); and (3) they use either sleeve (12), cuff-based (13), or end-to-end anastomosis. Mostly, BALB/C and C57BL/6J (B6) mice are used as donors and recipients, respectively, and sometimes in other combinations, such as male donors and female recipients. In the majority of cases, no immunosuppression is applied, generating a phenotype partially based on acute cellular rejection.

The combination of these multiple techniques raises the question of the comparability of the data generated. For example, the mechanisms underlying intimal hyperplasia are largely impacted by the severity of the injury (13, 14), which can in turn be affected by the type of anastomosis. While one study showed that intimal hyperplasia rates were independent of the type of anastomosis in mice (15), the influence of the type of anastomosis on the cellular composition of the murine aortic graft is so far unexplored.

To address this question, we provide a comparative report of the cellular distribution within murine aortic grafts connected using cuff and sleeve anastomosis strategies. Our data reveal the deep impact of the type of anastomosis on the cellular structure of the graft, both in terms of structure and immune cell infiltration.

## 2 Material and methods

### 2.1 Aortic transplantation in mice

**Figure 1:**
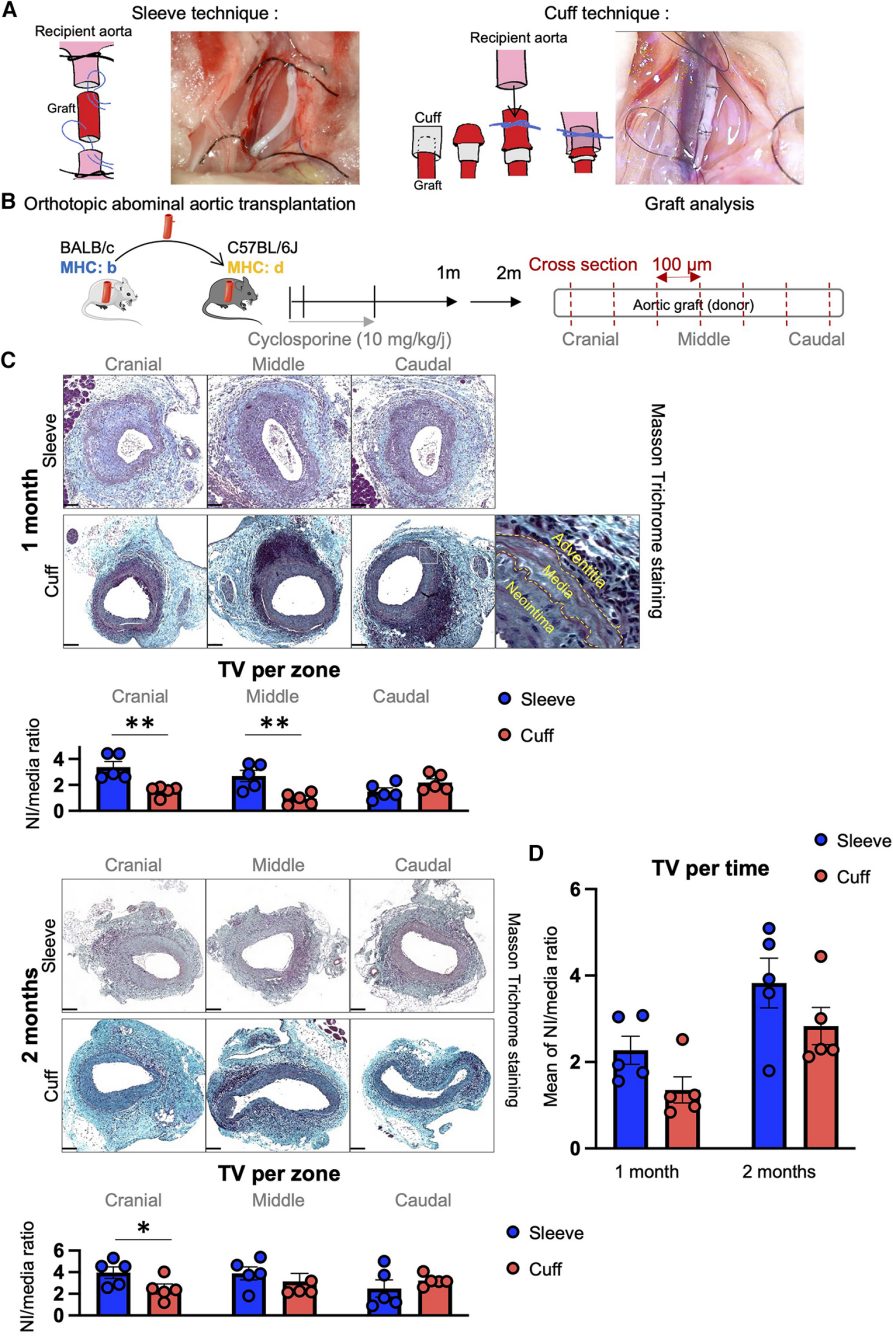
Induction of transplant vasculopathy after aortic transplantation in mice using the sleeve and cuff anastomosis methods. C57BL/6J recipient mice were orthotopically transplanted with abdominal aortas from BALB/C donor mice, using either the sleeve or the cuff anastomosis method and were analyzed at 1 and 2 months after transplantation (1 month: sleeve group *n* = 5, cuff group *n* = 5; 2 months: sleeve group, *n* = 5; cuff group: *n* = 5). (**A**) Schematic representation of the sleeve and cuff anastomosis methods and photomicrographs of the implantation into the recipient. (**B**) Experimental protocol time-line. Schematic representation of the histological analysis of the aortic grafts: 5 µm-thick cross-sections are cut every 100 µm along the aortic graft. (**C**) Representative images of histological cross-sections of aortic grafts from indicated groups and time-points stained with Masson Trichrome. Scale bar = 100 µm. Quantification of transplant vasculopathy using the neointima/media ratio in indicated zones of the graft. Data are represented as mean ± SEM and compared with Mann–Whitney test. *p < 0.05; **p < 0.01. (**D**) Average neointima/media ratio in the whole graft, regardless of the zones. Data are represented as mean ± SEM and compared with Mann-Whitney test.

In this study, BALB/C mice were used as donors and C57BL/6J as recipients. All mice were male, aged 8–12 weeks at the time of transplant, and purchased from ENVIGO RMS SARL (Harlan Laboratories, Gannat, France). The mice were housed at the animal facility (zootechnie de Rangueil, CREFRE, INSERM US006) under SPF conditions, at a regulated temperature (20 °C), with free access to food and water. Five donor mice and five recipient mice were used in each group for transplant surgeries. The surgical procedures are illustrated in Figure 1A.

#### 2.1.1 Pre- and perioperative care of donor and recipient mice

For both procedures (sleeve and cuff), the donor and recipient mice underwent the same pre- and perioperative care as follows. The mice were injected with buprenorphine (0.1 mg/kg, Buprécare, Centravet, #GTIN 03760087151893) and a cocktail of ketamine (50 mg/kg) (KETAMINE 1000, Virbac France, #03597132111010) and xylazine (10 mg/kg) (Rompun 2%, Centravet, #ROM001) 15 min before the start of the operation. In addition, they were maintained under gas anesthesia throughout the operation (isoflurane 0.5%–1.5%, O_2_ rate 0.2 L/min) (Vetflurane, Virbac France, VNR 137317; vaporizer: Medical Supplies and Services Int. Ltd., TemSega). The mice underwent the surgery on a heating pad (30 °C) and were covered with parafilm to prevent heat loss.

#### 2.1.2 Sleeve anastomosis procedure

##### 2.1.2.1 Aortic graft preparation from donor mice

Donor mice underwent a laparotomy. The internal organs covering the abdominal aorta (intestinal tract and genital organs) were retracted using a cotton swab and covered with saline-soaked gauze. The aorta was carefully dissociated from the inferior vena cava from the iliac to the mesenteric artery using fine forceps. All collaterals were ligated with 10-0 sutures and cut off. The 10-0 sutures used throughout the protocol were ETHILON #FG2850 (Johnson & Johnson). Once the abdominal aorta was exposed, the mouse underwent an intracardiac perfusion with heparinated phosphate buffered solution (PBS) and the aorta was harvested and kept at 4 °C in heparinated PBS until implantation. The donor mouse was euthanized under anesthesia by exsanguination followed by cervical dislocation.

##### 2.1.2.2 Implantation of donor aortic grafts into recipient mice using the sleeve anastomosis

Recipient mice underwent a laparotomy. The internal organs covering the abdominal aorta (intestinal tract and genital organs) were retracted using a cotton swab and covered with saline-soaked gauze. The aorta was carefully dissociated from the inferior vena cava from the iliac to slightly below the renal arteries using fine forceps. All collaterals were ligated with 10-0 monofilament sutures and cut off. The blood flow in the aorta was stopped using slipknots of 7-0 silk suture (MERSILK #FK803, Johnson & Johnson) placed below the renal arteries and above the iliac arteries. The exposed segment was transversally divided in the middle with micro-scissors. Both ends were rinsed with heparinized saline. The graft was placed in orthotopic position with the recipient aorta and the sleeve anastomosis was performed using 11-0 monofilament sutures (ETHILON #FG2881, Johnson & Johnson), being careful to avoid any torsion. The 7-0 slipknots were opened (first the caudal, followed by the cephalic knot) to restore blood flow. The absence of blood clot formation or bleeding was monitored for 2 min. The bowels and genital organs were replaced in the abdominal cavity and the peritoneal membrane and skin were closed using 6-0 sutures.

#### 2.1.3 Aortic transplantation in mice: cuff anastomosis procedure

##### 2.1.3.1 Aortic graft preparation from donor mice

Donor mice underwent a laparotomy. The internal organs covering the abdominal aorta (intestinal tract and genital organs) were retracted using a cotton swab and covered with saline-soaked gauze. The aorta was carefully dissociated from the inferior vena cava from the iliac to slightly below the renal arteries using fine forceps. All collaterals were ligated with 10-0 sutures and cut off. Once the abdominal aorta was exposed, the mouse underwent an intracardiac perfusion with heparinated PBS and the aorta was harvested. The donor mouse was euthanized under anesthesia by exsanguination followed by cervical dislocation. Homemade 24G Teflon cuffs were used (Intraflon2, PTFE 24G, L.17 mm—diameter 0.7 mm, Vygon #121.06). Both ends of the graft were pulled through a cuff and turned around to expose the endothelium. The cuffs were secured with two simple knots (10-0 monofilament sutures). The cuffed grafts were kept at 4 °C in heparinated PBS until implantation.

##### 2.1.3.2 Implantation of donor aortic grafts into recipient mice using the cuff anastomosis

Recipient mice underwent a laparotomy. The internal organs covering the abdominal aorta (intestinal tract and genital organs) were retracted using a cotton swab and covered with saline-soaked gauze. The aorta was carefully dissociated from the inferior vena cava from the iliac to slightly below the renal arteries using fine forceps. All collaterals were ligated with 10-0 monofilament sutures and cut off. The blood flow in the aorta was stopped using slipknots of 7-0 silk suture (MERSILK #FK803, Johnson & Johnson) placed below the renal arteries and above the iliac arteries. An incision was performed in the middle of the exposed segment using micro-scissors. The vessel was rinsed through the incision with heparinized saline. The graft was placed in an orthotopic position with the recipient aorta and the cuff anastomosis was performed and secured with 10-0 monofilament sutures, being careful to avoid any torsion. The 7-0 slipknots were opened (first the caudal, followed by the cephalic knot) to restore blood flow. The absence of blood clot formation or bleeding was monitored for 2 min. The bowels and genital organs were replaced in the abdominal cavity and the peritoneal membrane and skin were closed using 6-0 sutures.

#### 2.1.4 Postoperative care of recipient mice

Postoperatively, the recipient transplanted mice were allowed to recover in a cage placed on a heating pad and connected to O_2_ flow (1 L/min) until the next morning (12–16 h). The mice received buprenorphine injections immediately after the operation. All recipients were treated with intraperitoneal injections of cyclosporin A (10 mg/kg/day, #BML-195, Enzo Life Sciences): first, 1 h before transplantation, and then every day after the surgery for 14 days, in order to dampen the onset of acute rejection. The body condition of the mice was evaluated the next day. In case of paralysis of the hindlimbs (<10%), the mouse was euthanized. The surviving transplanted mice (90%) were then monitored every day until euthanasia.

### 2.2 Histology and morphometric analysis of mouse aortic grafts

Euthanasia of the recipient mice was achieved using a double method: overdose of anesthetics combined with exsanguination by intracardiac perfusion of heparinated PBS. The graft was collected, fixed in formol for 12 h, then embedded in paraffin. Cross-sections with a thickness of 4 μm were performed using a microtome (RM2145, Leica) and put on charged slides (Superfrost, ThermoFisher Scientific). For each vessel, sections were taken every 100 μm starting from the cephalic anastomosis until the caudal anastomosis. The obtained 100 μm-spaced sections were stained with Masson Trichrome and the slides were digitized using a slide scanner (3DHISTECH) set on brightfield 40× magnification. For each obtained image, areas of the lumen (A_lum_), and areas delimited by the internal and external elastic laminas (A_IEL_ and A_EEL_) were measured using the SlideViewer software (3DHISTECH). The area of the neointima was calculated as follows: A_NI_ = A_IEL_ − A_lum_; and the area of the media: A_M_ = A_EEL_ − A_IEL_. The extent of intimal hyperplasia was expressed as a A_NI_/A_M_ ratio in order to normalize the neointimal area.

### 2.3 Immunofluorescence of murine aortic grafts

Paraffin sections of aortic grafts (see Section 1.2) were deparaffinated by heating the slides at 60 °C for 10 min, soaking them in successive baths of xylene and ethanol, and then rehydrating them with water. The antigens were unmasked in citrate pH6 buffer using a pressure cooker. After cooling down, the sections were circled with a hydrophobic pen (DAKO). They were rehydrated in PBS, then PBS + Tween 0.1%. The unspecific binding was blocked by incubating the slides in PBS + BSA 3%. Then the slides were incubated at 4 °C overnight in a humid atmosphere with a solution of the primary antibody diluted in PBS + BSA 3% [primary antibodies: smooth muscle actin (SMA) rabbit polyclonal antibody, ThermoFisher, #12623297, 1:200; anti-mouse CD45 antibody, clone 30-F11, Biolegend #103101, 1:100; rabbit anti-CD3 antibody, clone SP7, Zytomed Systems #RBK024, 1:100; CD19 monoclonal antibody, clone LC1, ThermoFisher, #14-0190-82, 1:100; goat anti-mouse ICAM-1 antibody, clone M-19, Santa-Cruz Biotechnology, #sc-1511, 1:100; rabbit anti-mouse von Willebrand Factor (vWF) antibody, DAKO, #A0082, 1:100]. The slides were washed twice in PBS + Tween 0.1% before incubation with secondary antibodies (AF 488 Goat anti rabbit, Life Technologies, #A11034, 1:250; AF 555 Goat anti-mouse, Invitrogen, #A21452, 1:250; AF 555 Goat anti-rat, Invitrogen, #A214334, 1:250). After washing twice with PBS + Tween 0.1%, the slides were rinsed in distilled water and mounted with the ProLong Gold Antifade Mountant with DNA stain DAPI (ThermoFisher, #P36941). The immunofluorescence images were acquired using a confocal microscope (LSM900, Zeiss, TRI-Genotoul Imaging Facility, I2MC, Toulouse) and digitized with the ZEN software (Zeiss). The analysis was performed using ZEN and Fiji software. Smooth muscle cells, hematopoietic cells, T cells, and B cells, respectively, positive for αSMA, CD45, CD3, and CD19, were counted with the cell counter tool of Fiji. Fluorescence intensity measurements of ICAM-1 and vWF immunostaining in the luminal area were performed as follows. The process was implemented as a macro for the software the ImageJ/Fiji with the use of the MorpholibJ plugin (16). Using this plugin, we first applied a mask on the luminal layer (20 pixels width). Then, the mean intensity of the fluorescence signal (corresponding to ICAM-1 or vWF) was measured inside the mask, resulting in a quantification of the level of ICAM-1 or vWF protein content in the luminal (endothelial) layer.

### 2.4 Immunohistochemistry of murine aortic grafts

Paraffin sections of aortic grafts (see Section 1.2) were deparaffinated by heating the slides at 60 °C for 10 min, soaking them in successive baths of xylene and ethanol, and then rehydrating them with water. The antigens were unmasked in citrate pH6 buffer using a pressure cooker. After cooling down, the sections were circled with a hydrophobic pen (DAKO). They were rehydrated in PBS, then PBS + Tween 0.1%. Endogenous peroxidases were blocked with H_2_O_2_ (10 min) and the unspecific binding was blocked by incubating the slides in PBS + BSA 3%. The slides were then incubated at 4 °C overnight in a humid atmosphere with the primary antibody diluted in PBS + BSA 3% (primary antibody: monoclonal rat anti-mouse IP-10 antibody, R&D Systems, #MAB466, clone 134013, 1:50). The primary antibody was detected using an ImmPRESS HRP goat anti-rat IgG polymer detection kit (Vector Laboratories #MP-7404-50) followed by ImmPACT DAB Substrate Kit (Vector Laboratories #SK-4105). The images were acquired using a microscope (Leica DM2500) and the IP-10 signal was quantified using H&E DAB color deconvolution with Fiji software. Results are expressed as the percentage of positive pixels.

### 2.5 Quantification of circulating donor-specific antibodies

Blood samples from all mice were collected on the day of euthanasia in blood collection tubes (Sarstedt, #41.1395.005). The blood cell-free fraction was collected after centrifugation. For the analysis of donor-specific antibodies (DSA), plasma samples (diluted at 1:500, 1:1,000, and 1:10,000) were incubated for 30 min with splenocytes freshly isolated from BALB/C mice or from B6 mice (baseline negative control) and blocked with PBS + 10% fetal bovine serum in order to enable the binding of plasma antibodies directed against BALB/C epitopes. After washing, the antibody-bound splenocytes were incubated with an AF488-conjugated anti-mouse IgG antibody (Invitrogen #A1100) and a PE-Vio615 anti-mouse CD19 antibody (Miltenyi Biotech, #130-112-042) to exclude the non-specific detection of surface B-cell immunoglobulins. The data were acquired with a flow cytometer (LSRII BD Fortessa) and analyzed with the BD Diva software. The splenocyte-bound antibody levels were expressed as the mean fluorescence intensity (MFI) of AF488 on the population gated on live singlets after excluding CD19+ B cells. The MFI of B6-bound serum, considered to be an unspecific binding baseline, was subtracted to the MFI of BALB/C-bound serum to express the relative levels of DSA.

### 2.6 Study approval

The animal protocol for aortic transplantations was approved by the CEA 122 and the French Ministry of High Education and Research (Ministère de l’Enseignement Supérieur et de la Recherche) under number #28864.

### 2.7 Statistical analysis

The statistical significance of the data, presented as mean ± SEM, was evaluated using non-parametric Mann–Whitney *U* tests. Differences were considered statistically significant at *p* < 0.05.

## 3 Results

### 3.1 A progressive transplant vasculopathy develops in murine aortic grafts connected with both sleeve and cuff anastomosis

Aortic transplantation procedures using the cuff and sleeve anastomosis strategies were performed in male mice aged 8–12 weeks. A table recapitulating the ischemia times of both surgeries (Table 1) shows no statistical difference in the total and cold ischemia times between the sleeve and cuff groups but reveals a significantly longer warm ischemia time for the sleeve group. At 1 and 2 months after abdominal aortic transplantation, the aortic grafts were harvested and analyzed by histology (Figures 1A,B). Sections were taken every 100 μm starting from the cranial anastomosis and until the caudal anastomosis to provide an analysis of the full length of the graft and eventually identify anastomosis-dependent effects (Figure 1B). In both groups and at both time-points, the aortic graft cross-sections stained with Masson Trichrome displayed a large neointima composed of multiple cell layers and extracellular matrix (Figure 1C). The media appeared partially depleted of cells, indicating an immune reaction against the donor. The adventitia displayed extensive infiltrates of mononuclear cells, oftentimes organized as aggregates, suggesting an organization of immune cells in lymphoid follicles (Figure 1C). As the proximity to the anastomosis may affect the extent of intimal thickening (17, 18), we separated the graft into three zones for the quantitative analysis: cranial anastomosis; middle; and caudal anastomosis (Figures 1B,C). At 1 month post-transplantation, the proximity to the anastomosis indeed impacted the extent of intimal hyperplasia in the cuff group (cranial vs. middle, *p* = 0.056; caudal vs. middle, **p < 0.01). At 1 month post-transplantation, the extent of TV, expressed as the neointima/media ratio, was significantly higher in the sleeve vs. the cuff group in the cranial and middle areas (**p < 0.01, Mann–Whitney *U* test) (Figure 1C), reflected by significant differences in the lumen and neointima in the middle portions (Supplementary Figure S1). At 2 months, the neointima/media ratio was significantly higher in the sleeve vs. the cuff group in the cranial anastomosis portion (*p < 0.05, Mann–Whitney *U* test) (Figure 1C, Supplementary Figure S1). There were no major time-dependent effects of the average neointima/media ratio. The difference between 1 and 2 months in the cuff group was more noticeable than in the sleeve group (*p* = 0.056), suggesting that the development of TV in the cuff group was more progressive than in the sleeve group (Figure 1D).

**Table 1:** Ischemia times for murine aortic transplantation surgeries using either cuff or sleeve anastomosis.

	Cuff anastomosis	Sleeve anastomosis	
Total ischemia time (min ± SEM)	22.89 ± 4.02	28.60 ± 1.12	*p* = 0.1019
Cold ischemia time (min ± SEM)	12.56 ± 10.56	5.00 ± 0.00	*p* = 0.1029
Warm ischemia time (min ± SEM)	10.33 ± 1.236	23.60 ± 1.122	*p* = 0.0005***

***p < 0.001, Mann-Whitney test.

### 3.2 Neointimal VSMCs vs. hematopoietic cells in sleeve and cuff murine aortic grafts

To interrogate potential differences in the cellular composition of the grafts between the sleeve and cuff group, we performed a quantitative analysis of immunofluorescently stained aortic grafts.

**Figure 2:**
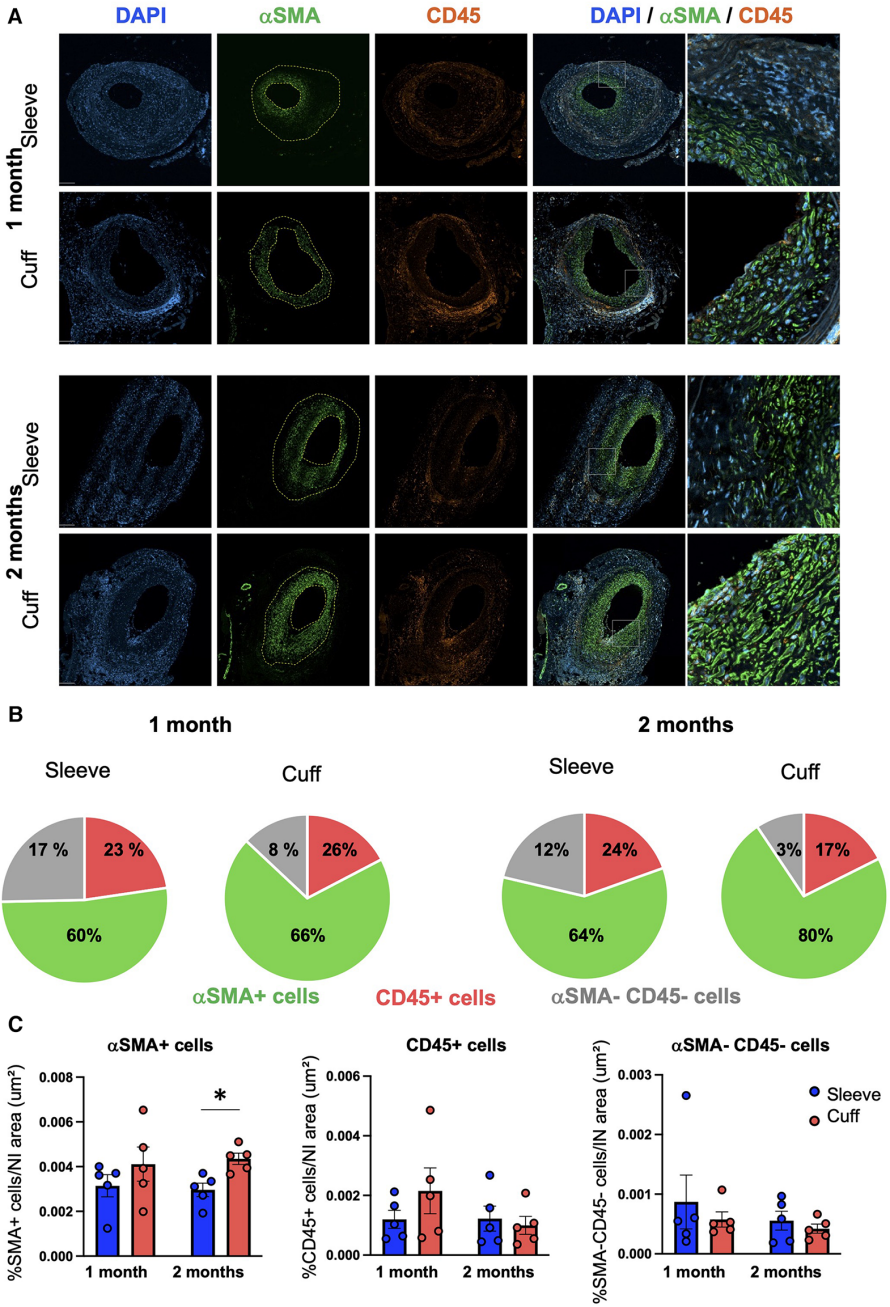
Impact of the sleeve and cuff anastomosis methods on the cellular composition of the neointima in aortic grafts. C57BL/6J recipient mice were orthotopically transplanted with abdominal aortas from BALB/C donor mice, using either the sleeve or the cuff anastomosis method and were analyzed at 1 and 2 months after transplantation (1 month: sleeve group *n* = 5, cuff group *n* = 5; 2 months: sleeve group, *n* = 5; cuff group: *n* = 5). (**A**) Representative immunofluorescence staining of aortic grafts of indicated groups and time-points. *α*SMA+ VSMCs appear in green, CD45+ hematopoietic cells in red and DAPI-stained nuclei in blue. The neointima is delimited in yellow. Scale bar = 100 µm. (**B**) Distribution of *α*SMA+ VSMCs and CD45+ hematopoietic cells within the neointima normalized by the total number of cells in indicated mice and time-points. (**C**) Quantification of *α*SMA+, CD45 + and *α*SMA−CD45− cells within the neointima normalized by the neointimal area in indicated mice. Data are represented as mean ± SEM and compared with Mann–Whitney tests. *p < 0.05.

We quantified VSMCs and hematopoietic cells inside the neointima based on their positivity for αSMA and CD45, respectively (Figure 2A). The numbers of neointimal αSMA-positive VSMCs, normalized to total neointimal cells (DAPI+), were higher on average in the cuff group vs. the sleeve group, as they represented 66% vs. 60% at 1 month, and 80% vs. 64% at 2 months, respectively (Figure 2B).

The distribution of neointimal VSMCs was different between the two groups: VSMCs populated the luminal layers of the neointima in the sleeve model, while they were evenly distributed throughout the whole neointimal layer in the cuff model (Figure 2A). To summarize this observation, we normalized the VSMC numbers to the neointimal area. The resulting neointimal VSMC coverage was significantly higher in the cuff group vs. the sleeve group at 2 months post-transplantation (*p < 0.05, Mann–Whitney *U* test) (Figure 2C). There were no major differences in either the CD45+ hematopoietic cells infiltrated within the neointima or in the numbers of αSMA-negative and CD45-negative cells between the cuff and sleeve groups (Figures 2B,C). No differences were observed when the various cell subsets were normalized to total DAPI+ cells, meaning that the difference observed came from the distribution of VSMCs throughout the neointimal area and not their overall proportion (Supplementary Figure S2).

These data indicate that the type of anastomosis is sufficient to impact the cellular composition and distribution inside the neointima during TV.

### 3.3 T- and B-cell infiltration differ in sleeve and cuff murine aortic grafts

TV, as a hallmark of chronic rejection, is characterized by the activation of the adaptive immune system against the graft. To explore the effect of the anastomosis on the types of lymphocytes found *in situ*, we quantified CD3+ T cells and CD19+ B cells on cross-sections of aortic grafts.

**Figure 3:**
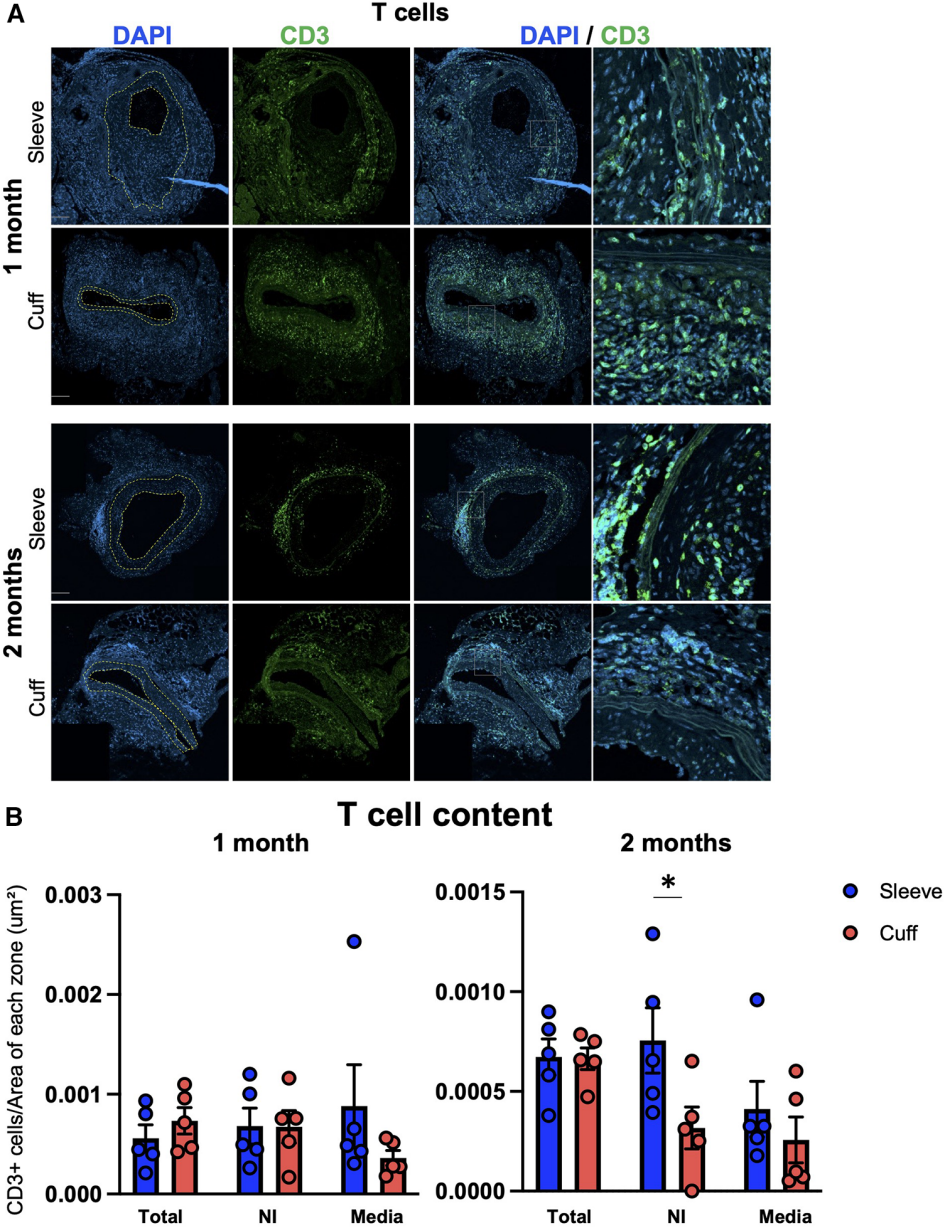
Impact of the sleeve and cuff anastomosis methods on T-cell infiltration of aortic grafts. C57BL/6J recipient mice were orthotopically transplanted with abdominal aortas from BALB/C donor mice, using either the sleeve or the cuff anastomosis method and were analyzed at 1 and 2 months after transplantation (1 month: sleeve group *n* = 5, cuff group *n* = 5; 2 months: sleeve group, *n* = 5; cuff group: *n* = 5). (**A**) Representative immunofluorescence staining of aortic grafts of indicated groups and time-points. CD3+ T cells appear in green and DAPI-stained nuclei in blue. The neointima is delimited in yellow. Scale bar = 100 µm. (**B**) T-cell content at 1 and 2 months within the total vessel section, neointima (NI) and media, normalized by the area of each zone in indicated mice. Data are represented as mean ± SEM and compared with Mann–Whitney tests. *p < 0.05.

T cells were observed in all three layers (neointima, media, adventitia) in grafts from both groups (Figure 3A). No major differences were observed in the total T-cell content per section, in the neointima or in the media in the cuff vs. the sleeve models at 1 month after transplantation (Figure 3B). At 2 months, however, higher numbers of T cells infiltrated the neointima in the sleeve group compared to the cuff group (*p < 0.05, Mann–Whitney *U* test) (Figure 3B).

**Figure 4:**
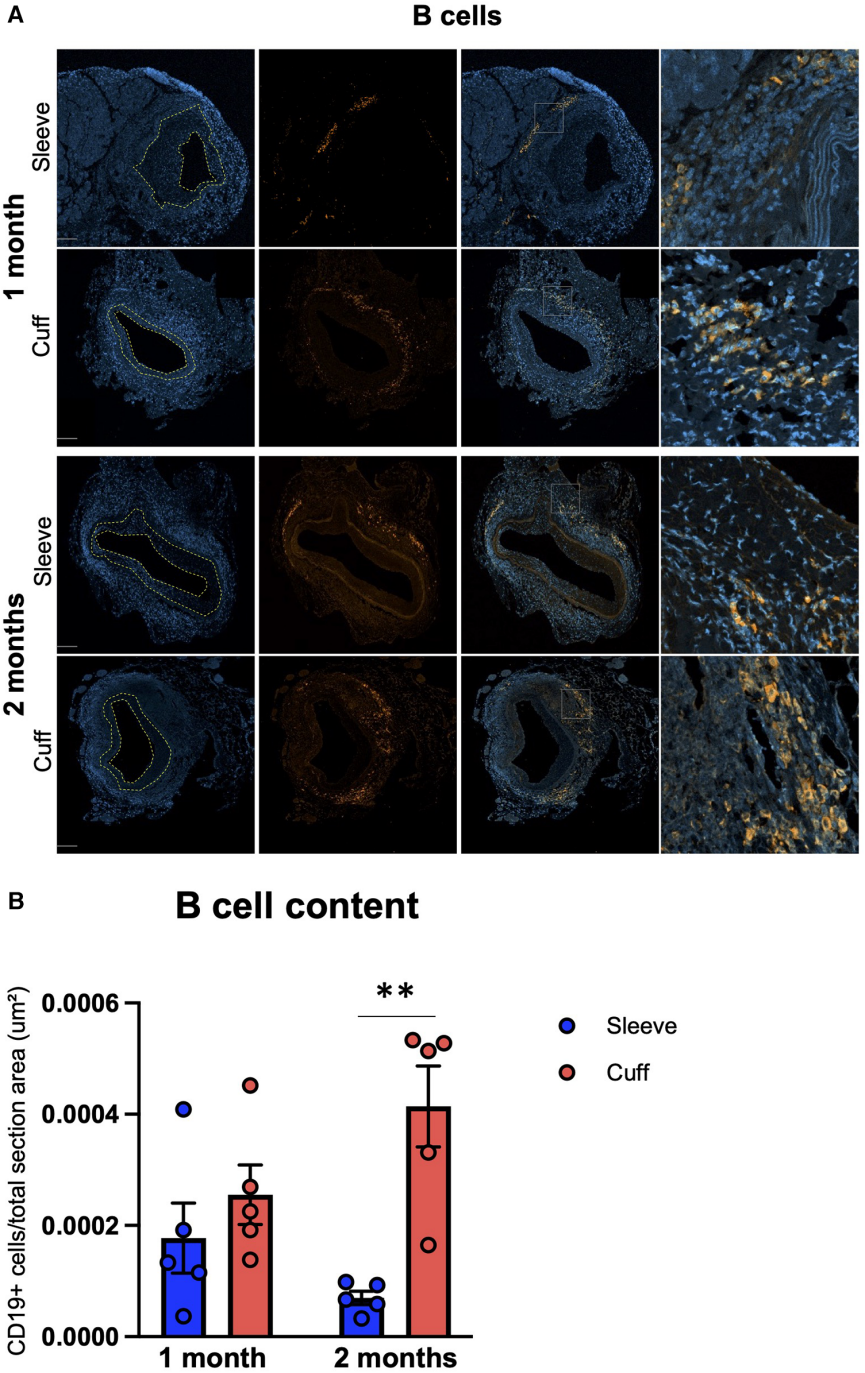
Impact of the sleeve and cuff anastomosis methods on B-cell infiltration of aortic grafts. C57BL/6J recipient mice were orthotopically transplanted with abdominal aortas from BALB/C donor mice, using either the sleeve or the cuff anastomosis method and were analyzed at 1 and 2 months after transplantation (1 month: sleeve group, *n* = 5; cuff group, *n* = 5; 2 months: sleeve group, *n* = 5; cuff group, *n* = 5). (**A**) Representative immunofluorescence staining of aortic grafts of indicated groups and time-points. CD19+ B-cells appear in green and DAPI-stained nuclei in blue. The neointima is delimited in yellow. Scale bar = 100 µm. (**B**) B-cell content at 1 and 2 months within total vessel, normalized to the total area of the vessel in indicated mice. Data are represented as mean ± SEM and compared with Mann–Whitney tests. **p < 0.01.

B cells were found in the adventitia in all groups (Figure 4A). They were either clustered together and intertwined with T cells, suggesting lymphoid aggregates, or disseminated along the external elastic lamina. This differential organization and variations in B-cell morphology (small round cells with a low cytosol/nucleus ratio inside the aggregates; larger cells with a higher cytosol/nucleus ratio in the disseminates fraction) suggest the coexistence of B-cell subpopulations inside the graft (as shown in Figure 4A, in the high magnification for the cuff group at 2 months). Unlike T cells, B cells were not observed in the neointima and the media (Figure 4A). Intriguingly, infiltrating B-cell numbers were significantly higher in the cuff group compared to the sleeve group at 2 months post-transplantation (**p < 0.01, Mann–Whitney *U* test) (Figure 4B). The differences observed in the B-cell numbers were not reflected by the quantification of systemic DSAs, whose levels did not show any significant differences between the sleeve and the cuff group (Supplementary Figure S3), suggesting and uncoupling between local intra-graft B cells and circulating antibodies.

**Figure 5:**
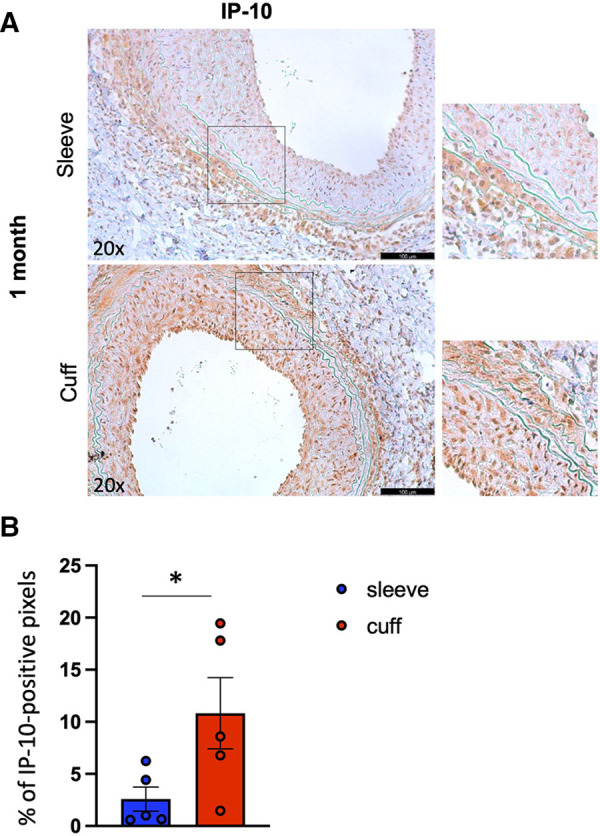
Impact of the sleeve and cuff anastomosis methods on local IP-10 abundance in aortic grafts. C57BL/6J recipient mice were orthotopically transplanted with abdominal aortas from BALB/C donor mice, using either the sleeve or the cuff anastomosis method and were analyzed at 1 month after transplantation (1 month: sleeve group *n* = 5, cuff group *n* = 5). (**A**) Representative immunohistochemical staining images (and zoomed areas) for chemokine IP-10 of aortic grafts of indicated groups and time-points. Scale bar = 100 µm. (**B**) CXCL10 content is expressed as % of positive pixels/section (brown signal) of indicated mice. Data are represented as mean ± SEM and compared with a Mann–Whitney test. *p < 0.05.

To better understand the processes underlying the differences observed between the two methods, we performed an immunofluorescence analysis of two markers of endothelial activation (ICAM-1, vWF) and an immunohistochemical analysis of the chemokine IP-10 (interferon-gamma-inducible protein 10 kDa), associated with graft rejection in patients, in particular antibody-mediated rejection (ABMR). At 1 month after aortic transplantation, the endothelial activation marker abundance in the endothelial layer was unchanged between the two methods (Supplementary Figure S4), while, interestingly, IP-10 abundance within the grafts was significantly higher when using the cuff vs. sleeve anastomosis (*p < 0.05, Mann–Whitney *U* test) (Figure 5).

Overall, our data uncovered a prominent effect of the type of anastomosis on the local immune profile of aortic grafts, associated with a differential content of the chemokine IP-10.

## 4 Discussion

In this study, we investigated how the type of anastomosis used for aortic transplantation in mice impacts the determinants of TV. While the type of anastomosis did not affect the overall histological features of TV, it deeply impacted the cellular landscape of aortic grafts.

We chose to compare the sleeve and the cuff anastomosis because in our experience both techniques are time-saving compared to the end-to-end anastomosis. Moreover, the sleeve anastomosis only involves one stitch, generating less trauma than the multiple stitches necessary to secure an end-to-end anastomosis. We used a BALB–B6 donor–recipient combination, as it is widely reported in the literature (13, 19). Choosing B6 as recipients presents the advantage of this strain being biased toward a Th1 response (20–22), typically associated with rejection in human transplant recipients (7, 23). Other mouse strain combinations are also found in the literature, such as male into female (24) or B6 into BALB, likely leading to different immune and inflammatory processes. To counteract the acute rejection due to a full major histocompatibility complex (MHC) mismatch between the two strains, and in an attempt to mimic the clinical situation, all recipient mice in our study were treated with a suboptimal dose of cyclosporin A to achieve immunosuppression during the first stages after transplantation (25). Oftentimes, no immunosuppression is used, likely increasing the severity of the trauma inflicted to the transplanted vessel wall (26). We transplanted abdominal aortas orthotopically to limit the bias due to altered blood flow. Both abdominal and thoracic aortas are used as donor tissue (27). Some groups use orthotopic transplantation and others connect the donor aorta to the carotid artery (13), submitting the vessel to a different hemodynamic constraint.

The detailed morphometric analysis of the aortic grafts, performed on multiple sections of each vessel, uncovered a more pronounced thickening of the vessel wall in the areas adjacent to the anastomosis at 1 month post-transplantation in the cuff group. This anastomosis effect on TV development is likely due to the severity of the trauma undergone by the vessel wall in the connecting areas (surgical manipulation, local changes in blood flow). In line with our data, Ni et al. show that the cellular processes underlying the formation of the neointima depend on the distance to anastomosis in a model of aortic transplantation into the carotid artery in mice (13).

VSMCs, identified by their positivity for αSMA, were the major cellular component of the neointima in both the cuff and sleeve groups. The higher VSMC coverage observed in the cuff vs. the sleeve group reveals the influence of the anastomotic method on the structure of the neointima. The percentages of VSMCs found in the cuff group clearly exceed those quantified after aorta-into-carotid transplantation in BALB to B6 mice without immunosuppression, suggesting that the trauma in this latter model leads to a more inflammatory neointima rich in recruited cells (19). Hematopoietic cells infiltrated the neointima in both groups, consistently with other studies (17, 28). It is still unclear, however, whether recruited cells contribute to VSMC coverage through differentiation or indirectly by releasing soluble mediators. While our study clearly highlights the quantitative predominance of VSMCs in the rejected vascular wall, little attention has so far been paid to their functions other than structural in TV development, as immune cells retained most of the focus. The high plasticity of VSMCs and their contribution to numerous vascular complications position them as an interesting candidate for further investigations in the context of TV. Besides VSMCs and hematopoietic cells, αSMA-CD45- cells were found in the neointima for the major part in the sleeve group. While we did not investigate the identity of these cells, we can hypothesize an endothelial or adventitial origin (14). Although the neointimal layer in human TV is commonly accepted as mainly composed of VSMCs, quantitative data specifying their proportion are unavailable. A study suggests that VSMCs are mostly observed in deeper layers of the neointima in human cardiac allograft vasculopathy (11), while the luminal layers contain mononuclear cells likely recruited from the circulation.

Our study was designed to provide a quantitative profile of VSMCs depending on the two different anastomosis types we used. Our methodology, however, does not enable us to draw any conclusion regarding the origins of neointimal VSMCs in TV, which has been a long-lasting debate. Both donor and recipient origins have been suggested for neointimal VSMCs in both mice and patients (17, 28, 29). The progenitors of neointimal VSMCs appear diverse and context-dependent: different studies suggest hematopoietic, adventitial, or medial origins (8, 13, 14, 30). Double reporter lineage tracing studies, already available in other contexts such as atherosclerosis or injury-induced restenosis but not TV, are necessary to put an end to this debate.

According to our data, the sleeve method is associated with a higher local T-cell content in aortic grafts than the cuff method, while the cuff model is distinguished by a more prominent accumulation of B cells at the later time-point. These results suggest that the two methods are not interchangeable and that their choice depends on the scientific question asked in each particular study. Transplant rejection historically relied on a T-cell-centric paradigm. As a consequence of the failure of T-cell-targeting therapies in significantly improving long-term outcomes after organ transplantation, B cells arose as key players in the context of chronic rejection over the recent years (32, 33). B cells are thought to participate to graft rejection through several mechanisms. Donor-specific antibodies produced by differentiated B cells are a major risk factor for chronic rejection, particularly for TV (2, 5, 34). Besides alloreactive B cells, autoreactive and polyreactive B-cell infiltrates are found in the adventitia of arteries with TV in cardiac allografts, suggesting a role for autoantibodies in transplant rejection (9). Interestingly, CD19+ B cells are specifically associated with arteries, in kidney allograft biopsies, independently on the rejection phenotype, as recently shown using spatial transcriptomics (35). Antibody-independent functions have also been highlighted, as B cells can provide help to alloreactive T cells (36) or could participate in maintaining an inflammatory milieu within the graft (37). Under some circumstances, B cells and antibodies are in contrast able to contribute to graft accommodation or tolerance (38, 39). The precise functions of B cells in transplant rejection are still under investigation. While the adaptive B-cell response is classically described in secondary lymphoid organs, multiple recent studies highlight the importance of graft-residing B cells in organ rejection after transplantation (32, 33, 37, 40). Graft-infiltrating B cells likely differ from their systemic counterparts by their phenotype and antibody repertoire (37), which can explain our seemingly controversial observation that while the B-cell content is higher in the cuff method graft, circulating DSA appear similar to the sleeve method. In the lung, local intra-graft B cells are responsible for ABMR (40). In general, the relative contribution of local vs. systemic B cells to graft rejection remains to be elucidated. The cuff model seems like an interesting option to investigate the role of local intra-graft B cells in rejection.

The different immune cell recruitment profiles observed between the two anastomosis methods could be explained by different blood flow patterns and warm ischemia times, respectively, induced by the cuff and sleeve anastomosis, which are likely to affect the behavior of endothelial cells (31) and subsequently immune cell infiltration kinetics. To address this possibility, we performed immunofluorescence staining for the adhesion molecule ICAM-1 and vWF, both reported as upregulated on the endothelium in contexts of inflammation (41–43). The lack of statistically significant difference in ICAM-1 and vWF content between the cuff and the sleeve group at 1 month post-transplantation (Supplementary Figure S4) suggests that the two methods impacted endothelial activation in similar ways.

To assess differences in cytokine/chemokine production between the two models, we performed immunostainings for the chemokine interferon-gamma-induced protein 10 kDa (IP-10, also known as CXCL10). Indeed, IP-10 is particularly interesting in the context of transplantation, because its increased levels are a common feature of the rejection of multiple transplanted organs in patients (heart (44), kidney (45), lung (46)), and this chemokine is heavily involved in vascular pathologies (47, 48), suggesting that it could play a role in TV development. There moreover seems to be a preferential association between IP-10 and the ABMR phenotype. Indeed, in the kidney transplantation context, the urinary IP-10:creatinine ratio improves the non-invasive diagnosis of ABMR and, when elevated, is associated with death-censored graft loss after ABMR (45). Finally, a group of transcripts including CXCL10 discriminates patients with ABMR after heart transplantation (44). Interestingly, a significantly higher IP-10 abundance was found in mouse aortic allografts in the cuff vs. the sleeve anastomosis group. This finding suggests that the cuff method is more suitable to mimic human transplant rejection, particularly ABMR, additionally characterized in the cuff group by a higher intra-graft B-cell infiltration compared with the sleeve group. The increase in IP-10 expression classically relies on the presence of interferon-gamma (49), suggesting that the immune response in the cuff model is Th1-driven.

Mouse models of aortic transplantation are not standardized. We highlight the deep impact of a minor modification of the experimental protocol on the outcomes of the graft and the underlying mechanisms, stressing the need to thoroughly characterize mouse models to estimate their relative suitability to address specific clinical questions.

## Data Availability

The raw data supporting the conclusions of this article will be made available by the authors, without undue reservation.
