# Supplementary material for: A minor tweak in transplant surgery protocols alters the cellular landscape of the arterial wall during transplant vasculopathy
Source: Front Transplant. 2024 Apr 29;3:1260125. doi: 10.3389/frtra.2024.1260125 (PMC11235260; doi:10.3389/frtra.2024.1260125)
Supplement: Supplementary file 1 [file Datasheet1.docx]

Supplementary Material

**SUPPLEMENTAL FIGURE 1:** Morphometric analysis of aortic graft according anastomosis technique and time post-transplantation


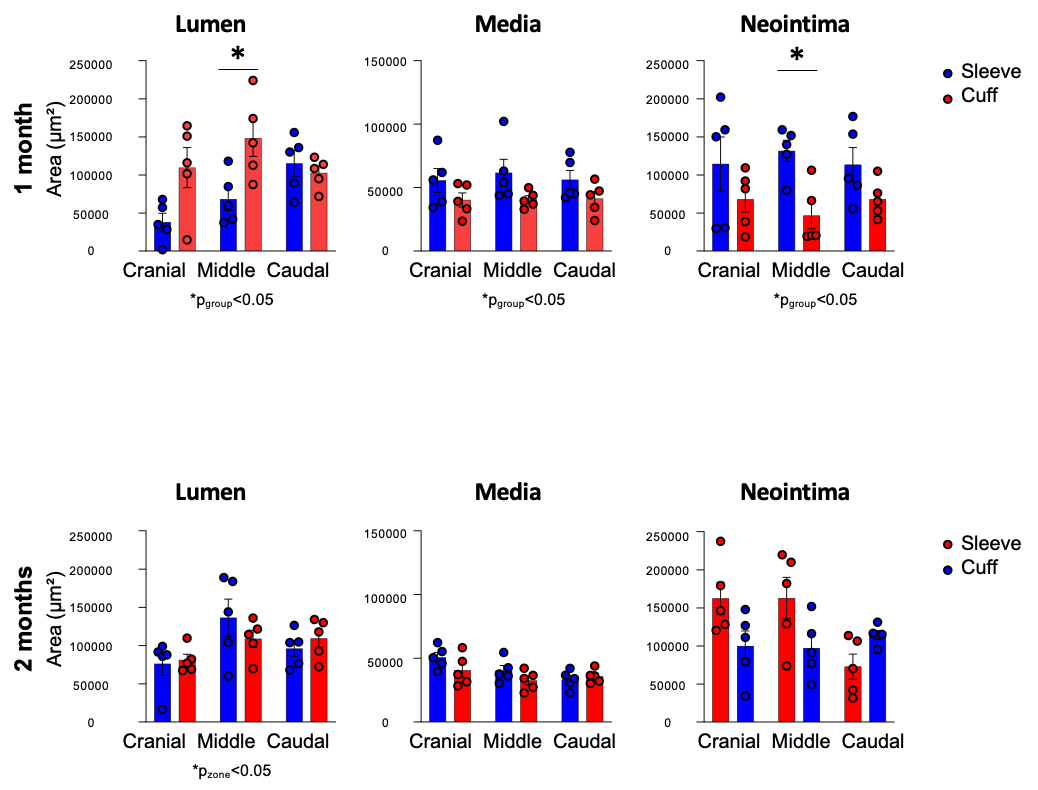


C57BL/6J recipient mice were orthotopically transplanted with abdominal aortas from BALB/C donor mice, using either the sleeve or the cuff anastomosis method and were analyzed at 1 and 2 months after transplantation (1 month: sleeve group n=5, cuff group n=5; 2 months: sleeve group, n=5; cuff group: n=5). Quantification of lumen, media and neointimal area of indicated mice at 1 and 2 months post-transplantation. Data are represented as mean +/- SEM and analyzed Mann-Whitney tests. *p<0.05.

**SUPPLEMENTAL FIGURE 2:** Composition of neointimal layer according anastomosis technique and time post-transplantation


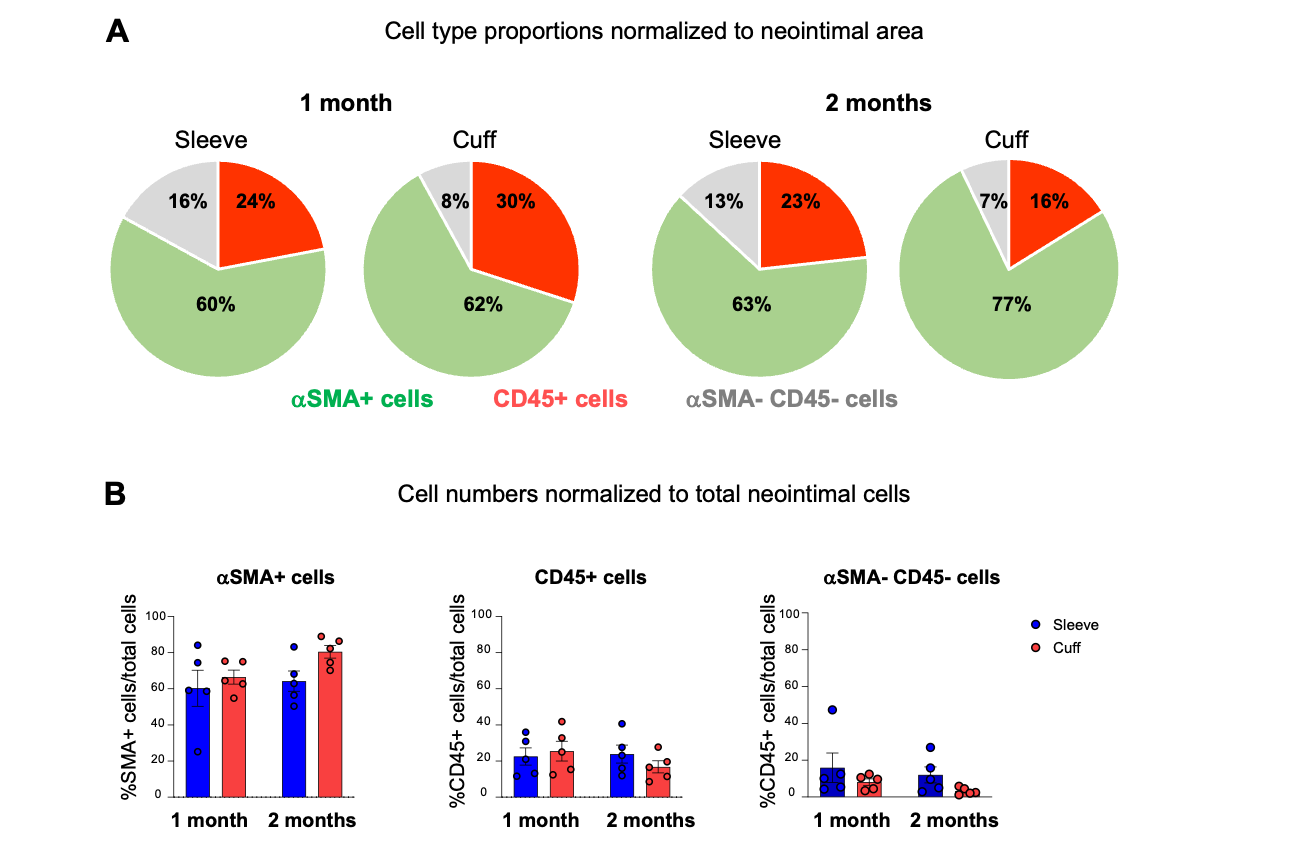


C57BL/6J recipient mice were orthotopically transplanted with abdominal aortas from BALB/C donor mice, using either the sleeve or the cuff anastomosis method and were analyzed at 1 and 2 months after transplantation (1 month: sleeve group n=5, cuff group n=5; 2 months: sleeve group, n=5; cuff group: n=5). **A**) Distribution of αSMA+ VSMCs and CD45+ hematopoietic cells within the neointima normalized to neointimal area in indicated mice and time-points. **B)** Quantification of αSMA+, CD45+ and αSMA-CD45- cells within the neointima normalized to total neointimal cells in indicated mice and time-points. Data are represented as mean +/- SEM and analyzed with Mann-Whitney tests.

**SUPPLEMENTAL FIGURE 3:** Circulating donor-specific antibody in transplanted mice depending on the anastomosis type


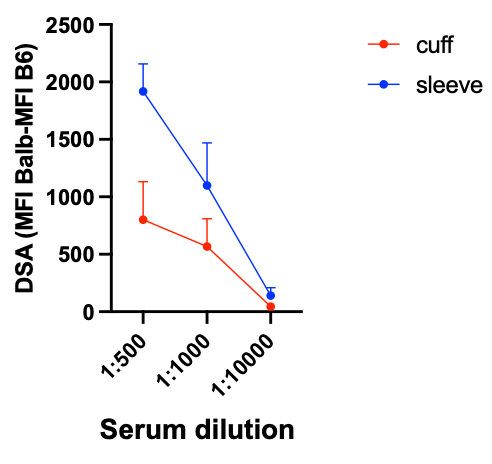


C57BL/6J recipient mice were orthotopically transplanted with abdominal aortas from BALB/C donor mice, using either the sleeve or the cuff anastomosis method and were analyzed at 2 months after transplantation (sleeve group, n=5; cuff group: n=5). Donor Specific Antibody levels were assessed by flow cytometry, using a splenocyte-serum binding assay. The level of DSA is expressed as the median fluorescence intensity (MFI) of serum-bound BALB/C splenocytes - median fluorescence intensity (MFI) of serum-bound B6 splenocytes (considered as a negative control). DSA levels are presented for the indicated dilutions of serum. The data, represented as mean +/- SEM were analyzed with Mann-Whitney test and no significance was found.

**SUPPLEMENTAL FIGURE 4:** Impact of the sleeve and cuff anastomosis on endothelial activation in aortic grafts


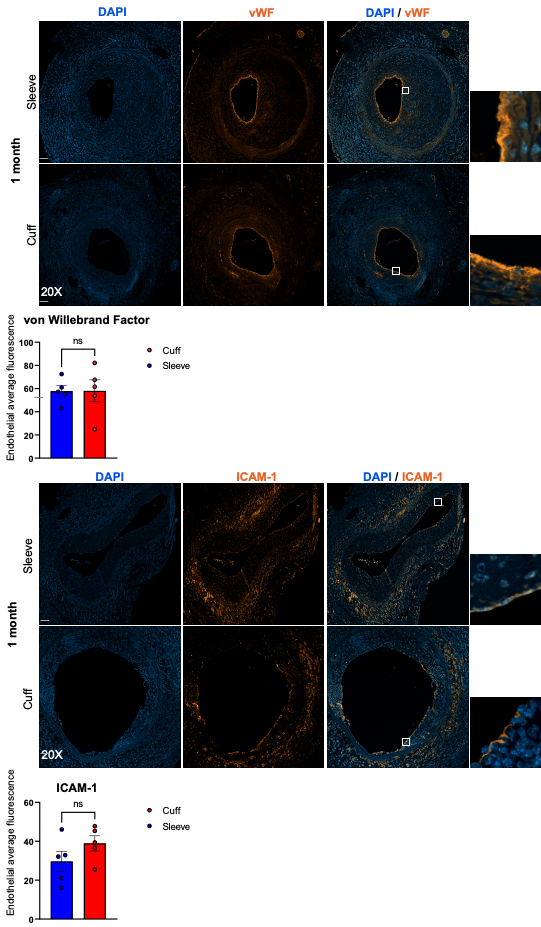


C57BL/6J recipient mice were orthotopically transplanted with abdominal aortas from BALB/C donor mice, using either the sleeve or the cuff anastomosis method and were analyzed at 1 month after transplantation (1 month: sleeve group n=5, cuff group n=5). Representative pictures of immunofluorescence staining for von Willbrand Factor (A) and ICAM-1 (B) of aortic grafts of indicated groups. vWF and ICAM-1 appear in red and DAPI-stained nuclei in blue. Scale bar = 100 µm. Data are represented as mean +/- SEM and compared with Mann-Whitney tests.

**
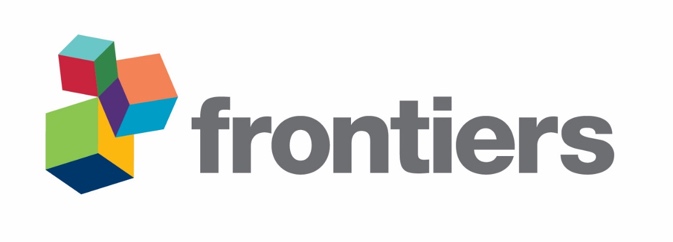
**
